# 14-Carbon demonstrates that some illegal ivory is being taken from government stockpiles

**DOI:** 10.1073/pnas.2211550119

**Published:** 2022-10-17

**Authors:** Thure E. Cerling, John E. Brown, Yves Hoareau, Paula Kahumbu, Tobias Odhacha, John R. Southon, Samuel K. Wasser

**Affiliations:** ^a^Department of Geology and Geophysics, University of Utah, Salt Lake City, UT 84112;; ^b^School of Biological Sciences, University of Utah, Salt Lake City, UT 84112;; ^c^Homeland Security Investigations, US Department of Homeland Security, Washington, DC 20536;; ^d^Center for Environmental Forensic Science, Department of Biology, University of Washington, Seattle, WA 98195-1800;; ^e^WildlifeDirect, Karen 00502, Nairobi, Kenya;; ^f^Earth System Science, University of California, Irvine, CA 92697-3100

**Keywords:** Africa, forensics, illegal wildlife trade, isotopes, ivory

## Abstract

The 14-carbon in animal tissues records the time that the tissues are formed; since the 1960s, using the “bomb curve” for ^14^C, the age of animal death can be determined accurately. Using animal tissue samples of known collection and formation dates for calibration, we determine the age of ivory samples from four ivory seizures made by law enforcement agencies between 2017 and 2019. The ^14^C measurements from these seizures show that most ivory in the illegal wildlife trade is from animals from recent poaching activities. However, one seizure has a large fraction of ivory that is more than 30 y old, consistent with markings on the tusks indicating they were derived from a government stockpile.

African elephant populations continue to be in decline due to poaching of elephants, primarily for their ivory, with a loss in population size of some 100,000 or more between 2007 and 2016 (1). Large volumes of ivory continue to be exported in shipping containers. Seventy percent of all ivory seizures are in volumes exceeding 0.5 tons, with an average size of over 2.5 tons (2). This begs the question of whether these large quantities of ivory are derived from recent poaching events or stockpiled ivory from elephants killed over decades, held by governments or transnational criminal organizations.

Bomb ^14^C measurements have been important in establishing tissue formation time for a variety of animal materials (3), including illegal materials such as cocaine (4) and ivory (5). We use the bomb ^14^C on seized African elephant ivory to estimate the lag time between animal death and time that authorities seized the ivory and therefore can evaluate whether the ivory is derived from recent poaching activities or is from stockpiled ivory stored either privately or by government agencies.

In this study, we analyze the most recently formed tissues in tusks from four large shipments of African elephant ivory seized in multiple countries between 2017 and 2019, to evaluate the lag time between animal death and seizure by law enforcement. We show that ivory continues to be derived from very recently killed animals with lag times of less than 3 y, but also that ivory for one seizure was primarily from elephants killed over 30 y ago with a very small distribution in ages. The latter results are consistent with markings on those tusks indicating they were derived from a reportedly well-guarded government stockpile.

## Results

### Calibration.

**Fig. 1.**
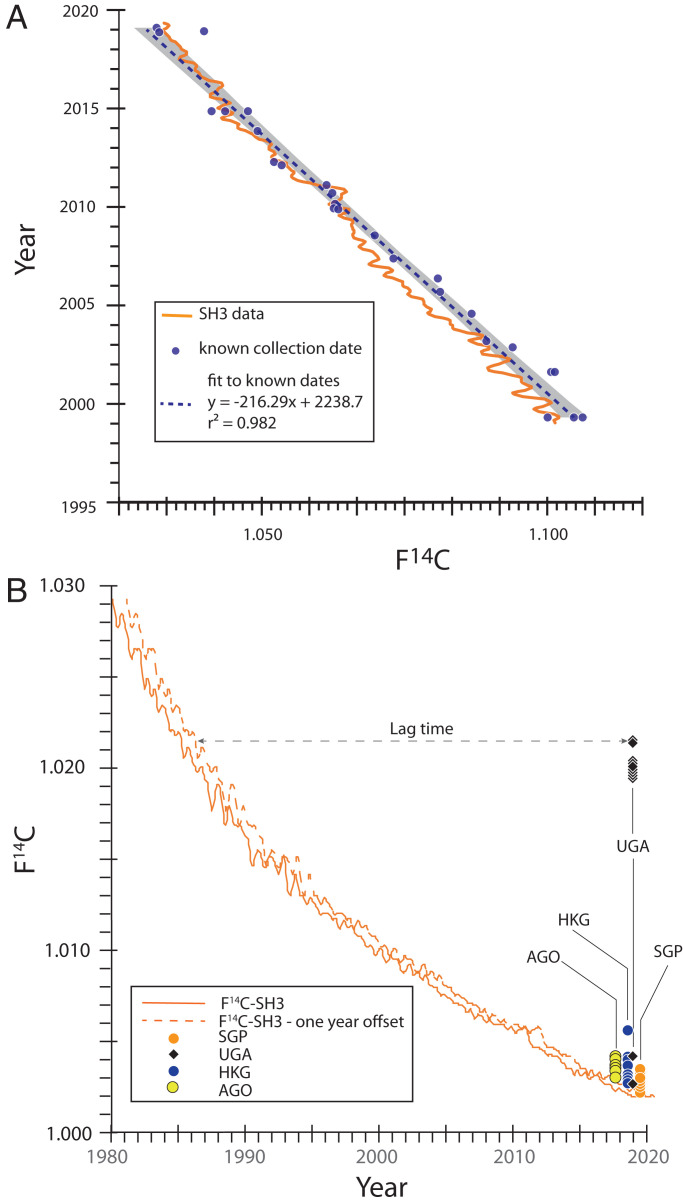
(*A*) F^14^C from tissues of known age from East Africa; data from Cerling et al. (5) and this study. Offset between the atmospheric SH3 curve and known tissue ages is 1.0 ± 1.0 y. (*B*) Age distribution of ivory samples from four ivory seizures made in 2017–2019. Lag time is the horizontal line between the data points and the 1-y offset of F^14^C for SH3.

The atmospheric decline in F^14^C since its peak in the early 1960s has been nearly linear for the past 20 y, although it is not linear over the entire time period since the 1960s (6); we compare our results to Southern Hemisphere Zone 3 (SH3) in the following discussion. Cerling et al. (5) showed a slight offset between the known tissue age of African samples and F^14^C for SH3 for the period from 2001 to 2013; therefore, we supplemented that dataset with samples collected in East Africa in 1999 and up to early 2019. Dataset S1 and Fig. 1*A* show the linear nature of the calibration for the period from 1999 to 2019; we use this calibration to calculate the time of death for the ivory samples in this study that are bracketed by this calibration interval. We note that the average offset in “age” between samples and the dates derived from https://c14.arch.ox.ac.uk/oxcal/OxCal.html (7) are offset by about 1 y. This is expected because mammals eat vegetation samples of a nonzero age from the time of photosynthesis, and also note that mammals recycle carbon, especially amino acids (8). We also use the calibration of the atmospheric F^14^C to compare to our calibrated samples and to estimate the time of death of samples that precede our calibration interval.

### Age Distribution in Recent Ivory Seizures.

**Fig. 2.**
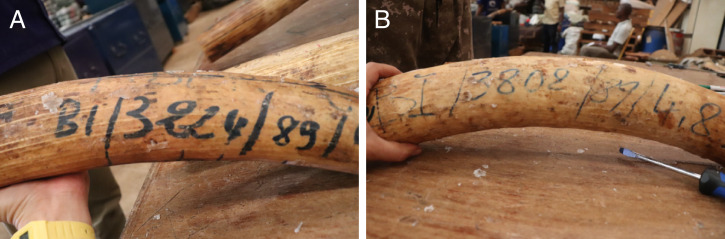
(*A*) Specimen with markings from the UG19A ivory seizure indicating country code/serial number/year/weight (in kilograms). See Parker (9) for discussion of CITES markings on the Burundi ivory stockpile. (*B*) Second specimen from UG19A ivory seizure.

This study focused on four ivory seizures made between 2017 and 2019: Angola (AGO, 22 August 2018, 1.8 tons), Hong Kong (HKG, 4 July 2017, 7.2 tons), Singapore (SGP, 21 July 2019, 8.8 tons), and Uganda (UGA, 24 January 2019, 3.3 tons). The UGA samples were noteworthy because a large portion of the ivory in that seizure included Convention on International Trade in Endangered Species of Wild Fauna and Flora (CITES) inventory markings indicating they were derived from the large Burundi government ivory stockpile (Fig. 2); that stockpile was inventoried and sealed in locked containers in 1989 (9). For three of the four seizures (AGO, HKG, and SGP), all samples had median lag times of less than 3 y (Dataset S2), with no samples having lag times greater than 10 y (Fig. 1*B*). These distributions are similar to our previous findings for 14 ivory seizures made between 2002 and 2014 comprising 231 analyses (5); the very short time between animal death and ivory seizure by law enforcement officials led to the conclusion that little “stockpile ivory” is entering the illegal wildlife trade, and the AGO, HKG, and SGP seizures fall into that pattern. However, the UGA ivory seizure has a very different distribution, with 9 of 11 samples examined having lag times of more than 30 y (Fig. 1*B*). The distribution in ages in the subset of nine samples is very small, with all samples having an estimated time of death between 1985 and 1988, just 1 y to 4 y before being cataloged by CITES in 1989; such a narrow distribution is suggestive that a highly controlled ivory stockpile, such as would be held by government bodies, was the source of these samples. Because the tusks in that stockpile have been reportedly secured by government authorities since that time, the stockpile should be reexamined to determine the extent of the breach and the potential of government involvement in the theft.

The original Burundi stockpile was inventoried and marked by CITES in 1986; most of this stockpile was exported to Belgium in late 1986. A second stockpile of 84 tons was cataloged by a CITES representative in 1989 with the added mission of determining whether the stockpile still consisted of old ivory. The representatives concluded that the majority of the 84 tons was old (10). However, our results suggest that a significant portion of the 84 tons may have been from elephants killed just 1 y to 4 y prior to being cataloged.

## Conclusions

Previous work using ^14^C showed that most ivory seized by law enforcement agencies was from animals that had recently been killed, presumably being poached for their ivory. One seizure from this study shows that most samples (*ca*. 90%) had lag times of about 30 y, suggesting long-term storage and likely from an existing government stockpile, based on CITES markings observed on some samples. This work suggests that some of the government stockpiles should be reexamined to evaluate their integrity.

## Methods

We supplemented the modern calibration dataset of Cerling et al. (5) with specimens of hair collected from East Africa over the period 1999–2019 to extend the earlier calibration curve; supplemental samples included both wildlife and domestic livestock. These additional hair samples were corrected by 0.33 y to account for time since tissue formation and collection.

Between 8 and 26 ivory samples were selected from four ivory seizures that were made between 2017 and 2019. Sampling of the first three seizures occurred independent of any physical features. However, sampling of the fourth seizure, described above as including tusks with CITES identifiers, included equal numbers of tusks with and without CITES markings. Genetic “fingerprints” (2, 11, 12) of all specimens were used as a guide to prevent repeat sampling of single individuals.

Dentine powder from ivory samples was collected and prepared as in Cerling et al. (5), except that collagen extraction and graphite target preparation were done at the W. M. Keck Carbon Cycle Accelerator Mass Spectrometry Laboratory at the University of California, Irvine, which made the radiocarbon measurements. Radiocarbon results are reported as fraction modern carbon (F^14^C) which specifically includes δ^13^C normalization to –25% using the Accelerator Mass Spectrometry measured δ^13^C value (13).

Results of the ^14^C measurements are presented in Datasets S1 and S2.

## Supplementary Material

Supplementary File

Supplementary File

## Data Availability

All study data are included in the article and/or supporting information. Previously published data were used for this work (5).
